# Supplementary material for: A versatile platform for precise synthesis of asymmetric molecular brush in one shot
Source: Nat Commun. 2017 Aug 24;8:333. doi: 10.1038/s41467-017-00365-2 (PMC5571111; doi:10.1038/s41467-017-00365-2)
Supplement: Supplementary file 1 — Supplementary Information [file 41467_2017_365_MOESM1_ESM.pdf]

Description of Supplementary Files

File Name: Supplementary Information
Description: Supplementary Figures, Supplementary Tables, Supplementary Methods and Supplementary References

File Name: Peer Review File

Supplementary Methods

Materials

2-(Dimethylamino)ether acrylate (DMAEA, Aldrich, 98%) and ethyl acrylate (EA, Aldrich, 99%) were passed through a basic alumina column to remove the stabilizer and distilled under reduced pressure from CaH_2 prior to use. Styrene (St, Aldrich, 99%) was washed with 5% aqueous NaOH solution and water, dried over $MgSO_4$, and distilled twice under reduced pressure from CaH_2 prior to use. Copper(I) bromide (CuBr, Aldrich, 98%) was purified by stirring overnight over CH_3COOH at room temperature, followed by washing with ethanol, diethyl ether, and acetone prior to drying at 40°C *in vacuo* for one day. 2,2'-Azobis(isobutyronitrile) (AIBN, Aldrich, 98%) was recrystallized from anhydrous ethanol twice. Cumyl dithiobenzoate (CDB),[1] PEO-N_3 (M_n = 775 and 2025 g/mol),[2] and pentafluorophenyl methacrylate (PFMA)[3] were synthesized according to previous reports. Triethylamine (TEA, Aldrich, 99.5%) was dried over KOH and distilled from CaH_2 under N_2 prior to use. Tetrahydrofuran (THF, Aldrich, 99%), dichloromethane (Aldrich, 99.5%), and toluene (Aldrich, 99%) were dried over CaH_2 and distilled from sodium and benzophenone under N_2 prior to use. *tert*-Butyl acrylate (*t*BA, Aldrich, 98%), 4-di(methylamino)pyridine (DMAP, Aldrich, 99%), formalin (Aldrich, 38 wt %), trifluoroacetic acid (TFA, Aldrich, 99%), 2-bromopropionic acid (Aldrich, 99%), *N*,*N*'-dicyclohexylcarbodiimide (DCC, Aldrich, 99%), 1,1,4,7,10,10-hexamethyl-triethylenetetramine (HMTETA, Aldrich, 97%), *N*,*N*,*N*',*N*',*N*''-pentamethyl-diethylenetriamine (PMDETA, Aldrich, 99%), 1,4-diazabicyclo[2.2.2]octane

(DABCO, Aldrich, 98%), *N*,*N*-diisopropylethylamine (DIPEA, Aldrich, 98%), propargyl bromide (Aladdin, 98%), benzylamine (Aldrich, 95%), and *N*,*N*-dimethylformamide (DMF, TCI, >99.5%) were used as received.

Instrumentation

All NMR analyses were performed on a Bruker Avance 500 spectrometer (500 MHz) in $CDCl_3$ and CD_2Cl_2; tetramethylsilane (^{1}H NMR) and $CDCl_3$ (^{13}C NMR) were used as internal standards, and CF_3CO_2H was used as external standard for ^{19}F NMR. FT-IR spectra were recorded on a Nicolet AVATAR-360 spectrophotometer with a 4 cm^{-1} resolution. High resolution mass spectrometry (HR MS) analysis was conducted on a Thermo Fisher Scientific LTQ FT Ultra mass spectrometer. Relative molecular weights and molecular weight distributions were measured by a conventional gel permeation chromatography (GPC) system equipped with a Waters 1515 Isocratic HPLC pump, a Waters 2414 refractive index detector and a set of Waters Styragel columns (HR3 (500-30,000), HR4 (5,000-600,000), and HR5 (50,000-4,000,000), 7.8×300 mm, particle size 5 μm). GPC measurements were carried out at 35 ℃ using THF as eluents with a flow rate of 1.0 mL/min. The system was calibrated with linear poly(methyl methacrylate) standards. GC analyses were performed on a Shimadzu GC-2014 spectrometer with a flame ionization detector (FID), using rtx-1 capillary column for the separation.

Synthesis of *tert*-butyl (2-hydroxymethyl)acrylate

*t*BA (20.5 g, 0.16 mol), DABCO (1.8 g, 0.016 mol), and TEA (1.7 g, 0.016 mol) were dissolved in 25 mL of THF followed by adding formalin (20.8 g, 0.25 mol) and water (15 mL). The solution was stirred at room temperature for 3 h followed by stirring at 55°C for 24 h. The aqueous phase was extracted by diethyl ether, and all organic layers were merged and washed by brine followed by drying over anhydrous Na_2SO_4. The filtrate was concentrated and distilled under reduced pressure to give 18.1 g (71.5%) of colorless liquid. ^{1}H NMR (500 MHz, $CDCl_3$): δ (ppm): 1.49 (s, 9H), 2.18 (s, 1H), 4.28 (s, 2H), 5.74 (d, J = 0.9 Hz, 1H), 6.15 (d, J = 0.9 Hz, 1H).

Synthesis of *tert*-butyl 2-((2-bromopropanoyloxy)methyl)acrylate 1

tert-Butyl (2-hydroxymethyl)acrylate (15.8 g, 0.1 mol), 2-bromopropionic acid (15.3 g, 0.1 mol), and DMAP (122.2 mg, 1 mmol) were dissolved in 200 mL of dry CH_2Cl_2. The mixture was cooled to 0°C before DCC (20.6 g, 0.1 mol) was added. The system was stirred at 0°C for 1 h, and was then warmed to room temperature with stirring overnight followed by filtration. A colorless liquid, *tert*-butyl 2-((2-bromopropanoyloxy)methyl)acrylate (*t*BBPMA) **1** (25.1 g, 85.6%), was obtained by silica column chromatography (eluent: ethyl acetate/hexane, v:v = 1:50). ^{1}H NMR (500 MHz, $CDCl_3$): δ (ppm): 1.49 (s, 9H), 1.85 (d, J = 6.9 Hz, 3H), 4.42 (q, J = 7.0 Hz, 1H), 4.82 (d, J = 13.9 Hz, 1H), 4.90 (d, J = 13.9 Hz, 1H), 5.82 (d, J = 0.9 Hz, 1H), 6.30 (d, J = 0.8 Hz, 1H).

Synthesis of 2-((2-bromopropanoyloxy)methyl)acrylic acid 2

*t*BBPMA **1** (3 g, 10.23 mmol) and dry CH_2Cl_2 (100 mL) were added to a 250 mL round bottom flask. The solution was stirred at 0°C for 30 min followed by adding TFA (35 g, 307 mmol) and the reaction mixture was warmed to 25°C. After stirring at room temperature for 4 h, the solution was concentrated and viscous solid was obtained after drying *in vacuo*. The crude product of 2-((2-bromopropanoyloxy)-methyl)acrylic acid **2** was used without further purification.

Synthesis of prop-2-yn-1-yl 2-((2-bromopropanoyloxy)methyl)acrylate 3

Propargyl bromide (2.1 g, 17.1 mmol) was first added to a 100 mL Schlenk flask (flame-dried under vacuum prior to use) sealed with a rubber septum for degassing and kept under N_2. A solution of 2-((2-bromopropanoyloxy)methyl)acrylic acid **2** (2.7 g, 11.4 mmol) in 30 mL of DMF were added. The reaction mixture was stirred at 0°C for 20 min followed by adding Na_2CO_3 (4.9 g, 46 mmol) and the reaction mixture was warmed to 25°C with stirring for 4 h. The reaction was quenched by adding $NaHCO_3$ aqueous solution (20 mL). The aqueous phase was extracted by CH_2Cl_2, and all organic layers were merged. The combined organic extracts were washed with brine three times, dried over $MgSO_4$, and concentrated. The residue was purified by silica column chromatography (eluent: ethyl acetate/hexane, v:v = 1:30) to afford 623 mg of prop-2-yn-1-yl 2-((2-bromopropanoyloxy)methyl)acrylate (Br-acrylate-alkyne, BAA) **3**. The total yield of two step reactions from *t*BBPMA **1** is 23.0%. FT-IR (KBr): ν (cm^{-1}): 3292, 3090, 2954, 2924, 2130, 1743, 1643, 1447, 1379, 1337, 1299, 1268,

1220, 1148, 1098, 1065, 1007, 811. ^{1}H NMR (500 MHz, $CDCl_3$): δ (ppm): 1.86 (d, J = 6.9 Hz, 3H), 2.50 (t, J = 2.4 Hz, 1H), 4.42 (q, J = 6.9 Hz, 1H), 4.80 (d, J = 2.5 Hz, 2H), 4.90 (d, J = 13.9 Hz, 1H), 4.96 (d, J = 13.9 Hz, 1H), 6.00 (d, J = 0.9 Hz, 1H), 6.48 (d, J = 0.7 Hz, 1H). ^{13}C NMR (125 MHz, $CDCl_3$): δ (ppm): 21.6 ($\mathit{C}H_3CHBr$), 39.8 ($CH_3\mathit{C}HBr$), 52.6 ($CH_2{=}CCO_2\mathit{C}H_2C{\equiv}CH$), 63.4 ($CH_2{=}C\mathit{C}H_2O_2C$), 75.8 ($CH_2{=}CCO_2CH_2C{\equiv}\mathit{C}H$), 77.5 ($CH_2{=}CCO_2CH_2\mathit{C}{\equiv}CH$), 129.0 ($\mathit{C}H_2{=}C$), 134.1 ($CH_2{=}\mathit{C}$), 164.1 ($CH_2{=}CCH_2O_2\mathit{C}$), 169.5 ($CH_2{=}C\mathit{C}O_2CH_2C{\equiv}CH$). HRMS (m/z): calcd for $C_{10}H_{12}BrO_4$ $[M+H]^+$: 274.9913, found: 274.9911.

RAFT homopolymerization of trifunctional acrylate monomer 3

In a typical procedure, AIBN (5.0 mg, 0.03 mmol) and CDB (25.0 mg, 0.09 mmol) were first added to a 10 mL Schlenk flask (flame-dried under vacuum prior to use) sealed with a rubber septum for degassing and kept under N_2. Next, BAA **3** (0.75 g, 2.7 mmol), and dry toluene (0.8 mL) were added via a gastight syringe. The flask was degassed by three cycles of freeze-pump-thaw followed by immersing the flask into an oil bath set at 70°C. The polymerization was terminated by immersing the flask into liquid N_2 after 20 h. The solution was precipitated into cold *n*-hexane. The crude product was purified by repeated dissolution and precipitation followed by drying *in vacuo* overnight to give 0.47 g of pink powder with a yield of 62.7%. Finally, AIBN was used to remove the dithiobenzoate moiety of the polymer at 65°C in THF according to a previous report.[4] 0.41 g of white powder, bifunctional macro-agent of poly(Br-acrylate-alkyne) (PBAA) **4b**, was obtained after drying *in vacuo* overnight.

GPC: M_n = 7,900 g/mol, M_w/M_n = 1.30. FT-IR (KBr): ν (cm^{-1}): 3288, 2957, 2927, 2127, 1742, 1645, 1452, 1265, 1157, 1091, 796, 686. ^1H NMR (CD_2Cl_2): δ (ppm): 1.91 (3H, CH_3CHBr), 2.13 (2H, CH_2C), 2.62 (1H, $CH_2CCO_2CH_2C{\equiv}CH$), 4.25 (1H, CH_3CHBr), 4.52 (2H, $CH_2CCO_2CH_2C{\equiv}CH$), 4.63 (2H, $CH_2CCH_2O_2C$), 7.15-7.36 (5H, phenyl). ^{13}C NMR ($CDCl_3$): δ (ppm): 22.0 (CH_3CHBr), 27.4 ($CH_2CCH_2O_2C$), 38.0 ($CH_2CCH_2O_2C$), 40.1 (CH_3CHBr), 48.2 ($CH_2CCO_2CH_2C{\equiv}CH$), 53.2 ($CH_2CCH_2O_2C$), 61.4 ($CH_2CCO_2CH_2C{\equiv}CH$), 66.7 ($CH_2CCO_2CH_2C{\equiv}CH$), 169.7 ($CH_2CCH_2O_2C$), 172.2 ($CH_2CCO_2CH_2C{\equiv}CH$).

Synthesis of PA-*g*-PEA/PEO 5 asymmetric molecular double-brush

CuBr (9 mg, 0.058 mmol) and PBAA **4b** ($M_{n,GPC}$ = 7,900 g/mol, M_w/M_n = 1.30, 8 mg, 0.029 mmol ATRP initiating group and alkynyl) were first added to a 25 mL Schlenk flask (flame-dried under vacuum prior to use) sealed with a rubber septum for degassing and kept under N_2. Next, EA (0.44 g, 4.35 mmol), PEO-N_3 (M_n = 775 g/mol, 90 mg, 0.116 mmol -N_3 group), PMDETA (15 μL, 0.058 mmol), and DMF (3 mL) were charged via a gastight syringe. The flask was degassed by three cycles of freeze-pump-thaw followed by immersing the flask into an oil bath set at 60°C. The polymerization and coupling reaction lasted 3 h and were terminated by immersing the flask into liquid N_2. The mixture was diluted by THF and passed through an alumina column to remove the residual copper catalyst. The solution was concentrated and precipitated into a mixture of hexane/diethyl ether (v:v = 2:1). After repeated purification by dissolving in THF and precipitating in hexane/diethyl ether five times

to completely remove the unreacted PEO-N_3, 27 mg of PA-*g*-PEA/PEO **5** asymmetric molecular double-brush was obtained after drying *in vacuo* overnight. GPC: M_n = 130,400 g/mol, M_w/M_n = 1.18. FT-IR (KBr): ν (cm^{-1}): 2957, 2926, 1732, 1448, 1378, 1259, 1159, 1098, 1025, 853, 797. ^{1}H NMR (CD_2Cl_2): δ (ppm): 0.85, 1.08, 1.21 (3H, $CO_2CH_2CH_3$), 1.45, 1.61, 1.87 (2H, CH_2C and 2H, CH_2CH), 2.24 (1H, CH_2CH), 3.31 (3H, OCH_3), 3.58 (4H, OCH_2CH_2), 4.07 (2H, $CO_2CH_2CH_3$, 2H, $CH_2CCH_2O_2C$, and 2H, $CH_2CCO_2CH_2$). ^{13}C NMR ($CDCl_3$): δ (ppm): 14.3 ($CO_2CH_2CH_3$), 35.6 (CH_2C and CH_2CH), 41.1 (CH_2CH), 60.7 ($CO_2CH_2CH_3$, $CH_2CCH_2O_2C$ and $CH_2CCO_2CH_2$), 70.6 (OCH_2CH_2), 174.6 (*C*=O).

ATRP kinetics was investigated by using PBAA **4b** ($M_{n,GPC}$ = 7,900 g/mol, M_w/M_n = 1.30) and the conversion of EA monomer was determined by GC, for which the content of EA monomer was calculated by using diphenyl ether as an internal standard. CuBr (9 mg, 0.058 mmol) and **4b** ($M_{n,GPC}$ = 7,900 g/mol, M_w/M_n = 1.30, 8 mg, 0.029 mmol ATRP initiating group) were first added to a 25 mL Schlenk flask (flame-dried under vacuum prior to use) sealed with a rubber septum for degassing and kept under N_2. Next, EA (0.44 g, 4.35 mmol), PEO-N_3 (M_n = 775 g/mol, 90 mg, 0.116 mmol -N_3 group), PMDETA (15 μL, 0.058 mmol), diphenyl ether (0.4 mL), and DMF (3 mL) were charged via a gastight syringe. The flask was degassed by three cycles of freeze-pump-thaw followed by immersing the flask into an oil bath set at 60°C. At appropriate time intervals, small aliquots were removed from the reaction mixture and placed in liquid N_2 to terminate the reaction. The quenched solution was characterized with GC analysis.

In a typical procedure, the efficiency of CuAAC click reaction was monitored by using PBAA **4b** ($M_{n,GPC}$ = 7,900 g/mol, M_w/M_n = 1.30) and the conversion of PEO-N_3 was determined by GPC, for which the content of PEO-N_3 before and after the reaction was calculated by using PS (M_n = 3,790 g/mol) as an internal standard, respectively. CuBr (9 mg, 0.058 mmol), PS (M_n = 3,790 g/mol) (13 mg), and **4b** ($M_{n,GPC}$ = 7,900 g/mol, M_w/M_n = 1.30, 8 mg, 0.029 mmol alkynyl) were first added to a 25 mL Schlenk flask (flame-dried under vacuum prior to use) sealed with a rubber septum for degassing and kept under N_2. Next, EA (0.44 g, 4.35 mmol), PEO-N_3 (M_n = 775 g/mol, 90 mg, 0.116 mmol -N_3 group), PMDETA (15 μL, 0.058 mmol), and DMF (3 mL) were charged via a gastight syringe. The flask was degassed by three cycles of freeze-pump-thaw followed by immersing the flask into an oil bath set at 60°C. A small aliquot was removed from the reaction mixture at appropriate time intervals (1 h, 2 h, and 3 h) and placed in liquid N_2 to terminate the reaction. The quenched solution was characterized with GPC analysis.

Synthesis of PA-*g*-PDMAEA/PEO 6 asymmetric molecular double-brush

CuBr (9 mg, 0.058 mmol) and PBAA **4b** ($M_{n,GPC}$ = 7,900 g/mol, M_w/M_n = 1.30, 8 mg, 0.029 mmol ATRP initiating group and alkynyl) were first added to a 25 mL Schlenk flask (flame-dried under vacuum prior to use) sealed with a rubber septum for degassing and kept under N_2. Next, DMAEA (0.83 g, 5.8 mmol), PEO-N_3 (M_n = 775 g/mol, 90 mg, 0.116 mmol -N_3 group), HMTETA (15 μL, 0.058 mmol), and DMF (3 mL) were charged via a gastight syringe. The flask was degassed by three cycles of

freeze-pump-thaw followed by immersing the flask into an oil bath set at 40°C. The polymerization and coupling reaction lasted 4 h and was terminated by immersing the flask into liquid N_2. The mixture was diluted by THF and passed through an alumina column to remove the residual copper catalyst. The solution was concentrated and precipitated into a mixture of hexane/diethyl ether (v/v = 2:1). After repeated purification by dissolving in THF and precipitating in hexane/diethyl ether five times to completely remove the unreacted PEO-N_3, 36 mg of PA-*g*-PDMAEA/PEO **6** asymmetric molecular double-brush was obtained after drying *in vacuo* overnight. GPC: M_n = 55,900 g/mol, M_w/M_n = 1.31. FT-IR (KBr): ν (cm^{-1}): 2950, 2862, 2821, 2770, 1733, 1455, 1398, 1262, 1162, 1099, 1042, 956, 801. ^{1}H NMR ($CDCl_3$): δ (ppm): 1.63, 1.90 (2H, CH_2C and 2H, CH_2CH), 2.25 (6H, $N(CH_3)_2$ and 1H, CH_2CH), 2.53 (2H, $CO_2CH_2CH_2N(CH_3)_2$), 3.36 (3H, OCH_3), 3.62 (4H, OCH_2CH_2), 4.13 (2H, $CO_2CH_2CH_2N(CH_3)_2$, 2H, $CH_2CCH_2O_2C$, and 2H, $CH_2CCO_2CH_2$). ^{13}C NMR ($CDCl_3$): δ (ppm): 35.1 (CH_2C and CH_2CH), 41.4 (CH_2CH), 45.8 ($N(CH_3)_2$), 57.6 ($CO_2CH_2CH_2N(CH_3)_2$), 62.3($CO_2CH_2CH_2N(CH_3)_2$, $CH_2CCH_2O_2C$ and $CH_2CCO_2CH_2$), 70.6 (OCH_2CH_2), 174.5 (C=O).

ATRP kinetics was investigated by using PBAA **4b** ($M_{n,GPC}$ = 7,900 g/mol, M_w/M_n = 1.30) and the conversion of DMAEA monomer was determined by GC, for which the content of DMAEA monomer was calculated by using diphenyl ether as an internal standard. CuBr (9 mg, 0.058 mmol) and **4b** ($M_{n,GPC}$ = 7,900 g/mol, M_w/M_n = 1.30, 8 mg, 0.029 mmol ATRP initiating group) were first added to a 25 mL Schlenk flask (flame-dried under vacuum prior to use) sealed with a rubber septum for

degassing and kept under N_2. Next, DMAEA (0.83 g, 5.8 mmol), PEO-N_3 (M_n = 775 g/mol, 90 mg, 0.116 mmol-N_3 group), HMTETA (15 μL, 0.058 mmol), diphenyl ether (0.8 mL), and DMF (3 mL) were charged via a gastight syringe. The flask was degassed by three cycles of freeze-pump-thaw followed by immersing the flask into an oil bath set at 40°C. At appropriate time intervals, small aliquots were removed from the reaction mixture and placed in liquid N_2 to terminate the reaction. The quenched solution was characterized with GC analysis.

The efficiency of CuAAC click reaction was monitored by using PBAA **4b** ($M_{n,GPC}$ = 7,900 g/mol, M_w/M_n = 1.30) and the conversion of PEO-N_3 was determined by GPC, for which the content of PEO-N_3 before and after the reaction was calculated by using PS (M_n = 3,790 g/mol) as an internal standard, respectively. CuBr (9 mg, 0.058 mmol), PS (M_n = 3,790 g/mol) (15 mg), and **4b** ($M_{n,GPC}$ = 7,900 g/mol, M_w/M_n = 1.30, 8 mg, 0.029 mmol alkynyl) were first added to a 25 mL Schlenk flask (flame-dried under vacuum prior to use) sealed with a rubber septum for degassing and kept under N_2. Next, DMAEA (0.83 g, 5.8 mmol), PEO-N_3 (M_n = 775 g/mol, 90 mg, 0.116 mmol -N_3 group), HMTETA (15 μL, 0.058 mmol), and DMF (3 mL) were charged via a gastight syringe. The flask was degassed by three cycles of freeze-pump-thaw followed by immersing the flask into an oil bath set at 40°C. A small aliquot was removed from the reaction mixture after 4 h and placed in liquid N_2 to terminate the reaction. The quenched solution was characterized with GPC analysis.

Synthesis of PA-*g*-PS/PEO 7 asymmetric molecular double-brush

CuBr (9 mg, 0.058 mmol) and PBAA **4b** ($M_{n,GPC}$ = 7,900 g/mol, M_w/M_n = 1.30, 8 mg, 0.029 mmol ATRP initiating group and alkynyl) were first added to a 25 mL Schlenk flask (flame-dried under vacuum prior to use) sealed with a rubber septum for degassing and kept under N_2. Next, St (0.61 g, 5.8 mmol), PEO-N_3 (M_n = 775 g/mol, 90 mg, 0.116 mmol -N_3 group), HMTETA (15 μL, 0.058 mmol), and DMF (3 mL) were charged via a gastight syringe. The flask was degassed by three cycles of freeze-pump-thaw followed by immersing the flask into an oil bath set at 80°C. The polymerization and coupling reaction lasted 4 h and was terminated by immersing the flask into liquid N_2. The mixture was diluted by THF and passed through an alumina column to remove the residual copper catalyst. The solution was concentrated and precipitated into a mixture of hexane/diethyl ether (v/v = 2:1). After repeated purification by dissolving in THF and precipitating in hexane/diethyl ether five times to completely remove the unreacted PEO-N_3, 31 mg of PA-*g*-PS/PEO **7** asymmetric molecular double-brush was obtained after drying *in vacuo* overnight. GPC: M_n = 67,900 g/mol, M_w/M_n = 1.28. FT-IR (KBr): ν (cm^{-1}): 3060, 3026, 2962, 2924, 2850, 1738, 1601, 1493, 1452, 1261, 1100, 1028, 800, 758, 699. ^{1}H NMR (CD_2Cl_2): δ (ppm): 1.45 (2H, CH_2C, and 2H, $C_6H_5CHCH_2$), 1.81 (1H, $C_6H_5CHCH_2$), 3.33 (3H, OCH_3), 3.59 (4H, OCH_2CH_2), 6.58, 7.05 (5H, $C_6H_5CHCH_2$). ^{13}C NMR ($CDCl_3$): δ (ppm): 30.2 (CH_2C and CH_2CH), 43.6 ($C_6H_5CHCH_2$), 59.2 (OCH_3), 66.0 ($CH_2CCH_2O_2C$ and $CH_2CCO_2CH_2$), 70.8 (OCH_2CH_2), 125.8, 128.1, 145.4 (C_6H_5), 174.8 (C=O).

ATRP kinetics was investigated by using PBAA **4b** ($M_{n,GPC}$ = 7,900 g/mol, M_w/M_n = 1.30) and the conversion of St monomer was determined by GC, for which the content of St monomer was calculated by using diphenyl ether as an internal standard. CuBr (9 mg, 0.058 mmol) and **4b** ($M_{n,GPC}$ = 7,900 g/mol, M_w/M_n = 1.30, 8 mg, 0.029 mmol ATRP initiating group) were first added to a 25 mL Schlenk flask (flame-dried under vacuum prior to use) sealed with a rubber septum for degassing and kept under N_2. Next, St (0.61 g, 5.8 mmol), PEO-N_3 (M_n = 775 g/mol, 90 mg, 0.116 mmol -N_3 group), HMTETA (15 μL, 0.058 mmol), diphenyl ether (0.6 mL), and DMF (3 mL) were charged via a gastight syringe. The flask was degassed by three cycles of freeze-pump-thaw followed by immersing the flask into an oil bath set at 80°C. At appropriate time intervals, small aliquots were removed from the reaction mixture and placed in liquid N_2 to terminate the reaction. The quenched solution was characterized with GC analysis.

Synthesis of PA-*g*-PPFMA/PEO 8 asymmetric molecular double-brush

CuBr (9 mg, 0.058 mmol) and PBAA **4b** ($M_{n,GPC}$ = 7,900 g/mol, M_w/M_n = 1.30, 8 mg, 0.029 mmol ATRP initiating group) were first added to a 25 mL Schlenk flask (flame-dried under vacuum prior to use) sealed with a rubber septum for degassing and kept under N_2. Next, PFMA (1.10 g, 4.35 mmol), PEO-N_3 (M_n = 775 g/mol, 90 mg, 0.116 mmol -N_3 group), PMDETA (15 μL, 0.058 mmol), and DMF (3 mL) were charged via a gastight syringe. The flask was degassed by three cycles of freeze-pump-thaw followed by immersing the flask into an oil bath set at 80°C. The

polymerization and coupling reaction lasted 5 h and was terminated by immersing the flask into liquid N_2. The mixture was diluted by THF and passed through an alumina column to remove the residual copper catalyst. The solution was concentrated and precipitated into a mixture of hexane/ether (v/v = 2:1). After repeated purification by dissolving in THF and precipitating in hexane/diethyl ether five times to completely remove the unreacted PEO-N_3, 23 mg of PA-*g*-PPFMA/PEO **8** asymmetric molecular double-brush was obtained after drying *in vacuo* overnight. GPC: M_n = 14,600 g/mol, M_w/M_n = 1.20. FT-IR (KBr): ν (cm^{-1}): 2950, 2861, 2821, 2770, 1732, 1455, 1398, 1262, 1162, 1099, 1042, 956, 801. ^{1}H NMR ($CDCl_3$): δ (ppm): 0.82, 1.24 (3H, CH_2CCH_3), 1.40, 1.60 (2H, CH_2CCH_3, and 2H, CH_2CCH_2), 3.36 (3H, OCH_3), 3.63 (4H, OCH_2CH_2). ^{19}F NMR ($CDCl_3$): δ (ppm): -153.2, -157.7, -162.2.

ATRP kinetics was investigated by using PBAA **4b** ($M_{n,GPC}$ = 7,900 g/mol, M_w/M_n = 1.30) and the conversion of PFMA monomer was determined by GC, for which the concentration of PFMA monomer was calculated by using diphenyl ether as an internal standard. CuBr (9 mg, 0.058 mmol) and **4b** ($M_{n,GPC}$ = 7,900 g/mol, M_w/M_n = 1.30, 8 mg, 0.029 mmol ATRP initiating group) were first added to a 25 mL Schlenk flask (flame-dried under vacuum prior to use) sealed with a rubber septum for degassing and kept under N_2. Next, PFMA (1.10 g, 4.35 mmol), PEO-N_3 (M_n = 775 g/mol, 90 mg, 0.116 mmol -N_3 group), PMDETA (15 μL, 0.058 mmol), diphenyl ether (1.0 mL), and DMF (3 mL) were charged via a gastight syringe. The flask was degassed by three cycles of freeze-pump-thaw followed by immersing the flask into an oil bath set at 80°C. At appropriate time intervals, small aliquots were removed

from the reaction mixture and placed in liquid N_2 to terminate the reaction. The quenched solution was characterized with GC analysis.

Preparation of PA-*g*-PBMAm/PEO 9 asymmetric molecular double-brush

PA-*g*-PPFMA/PEO **8** (15 mg) was dissolved in 5 mL of dry THF under N_2 followed by adding benzylamine (22 μL, 0.2 mmol) and DIPEA (35 μL, 0.2 mmol) via a gastight syringe. The reaction mixture was stirred at 50°C under N_2 for 10 h and then it was precipitated into a mixture of hexane/diethyl ether (v/v = 1:1) to remove free pentafluorophenol. The final product, 10 mg of PA-*g*-PBMAm/PEO **9** asymmetric molecular double-brush, was obtained by drying *in vacuo* overnight. GPC: M_n = 11,100 g/mol, M_w/M_n = 1.25. ^{1}H NMR ($CDCl_3$): δ (ppm): 0.85, 1.25 (3H, CH_2CCH_3), 1.78 (2H, CH_2CCH_2, and 2H, CH_2CCH_3), 3.32 (3H, OCH_3), 3.59 (4H, OCH_2CH_2), 4.26 (2H, $C_6H_5CH_2$, 2H, $CH_2CCH_2O_2C$, and 2H, $CH_2CCO_2CH_2$), 7.25 (5H, $C_6H_5CH_2$).

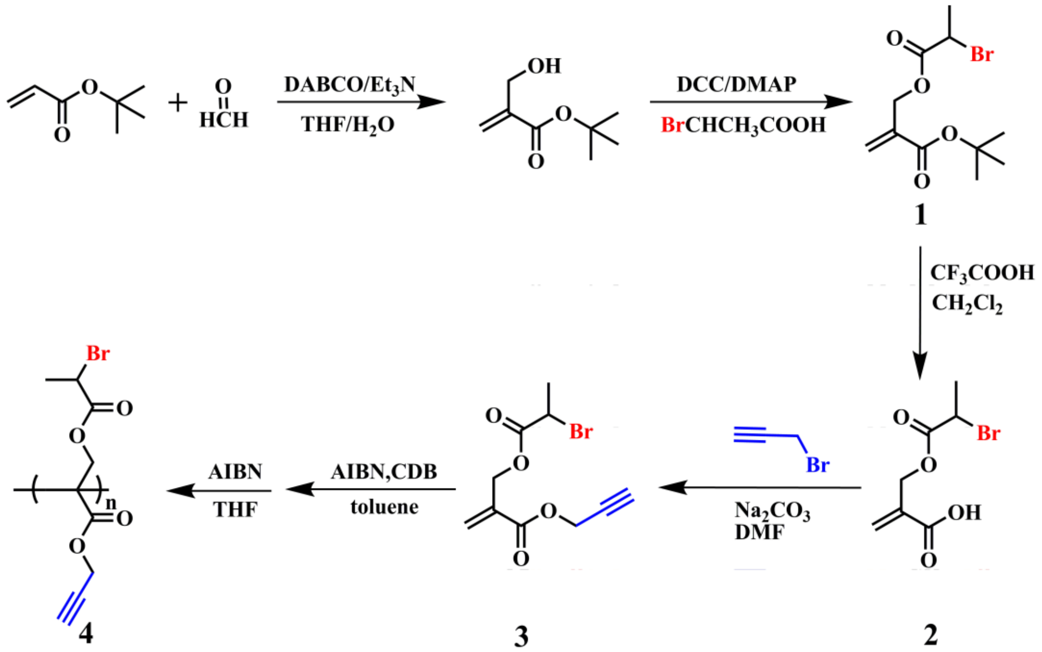

Supplementary Figure 1. Synthesis of Br-acrylate-alkyne **3** trifunctional monomer and PBAA **4** bifunctional macro-agent.

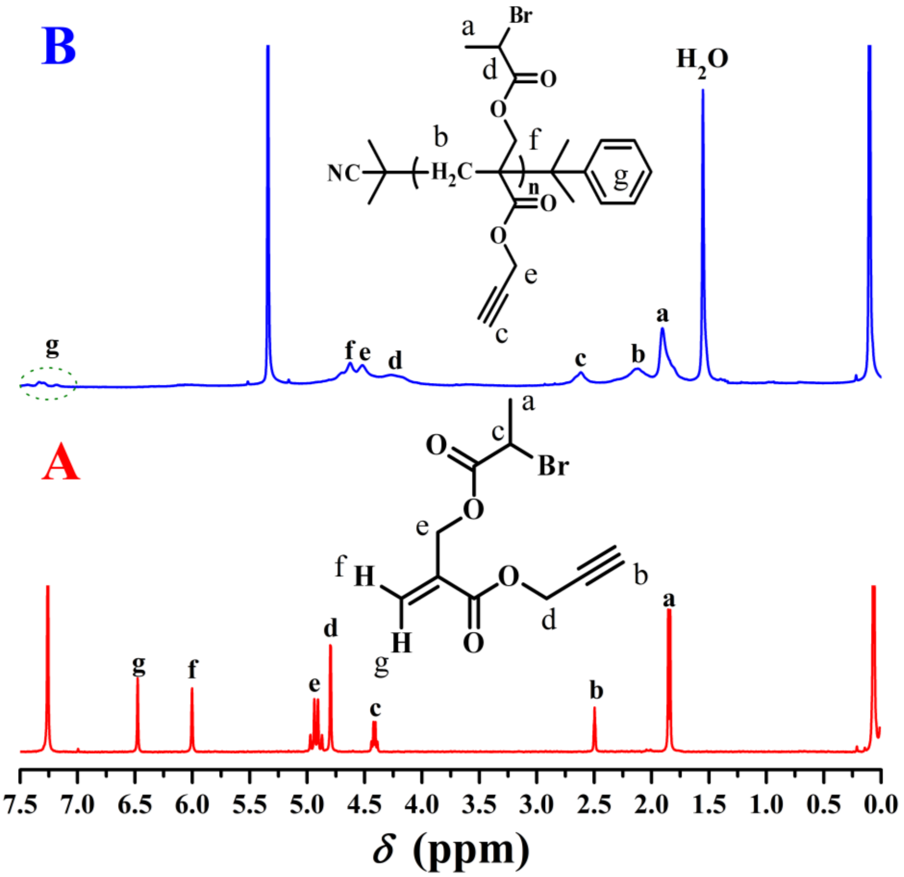

Supplementary Figure 2. ^{1}H NMR spectra of (A) Br-acrylate-alkyne **3** trifunctional monomer in $CDCl_3$ and (B) PBAA **4** macro-agent in CD_2Cl_2.

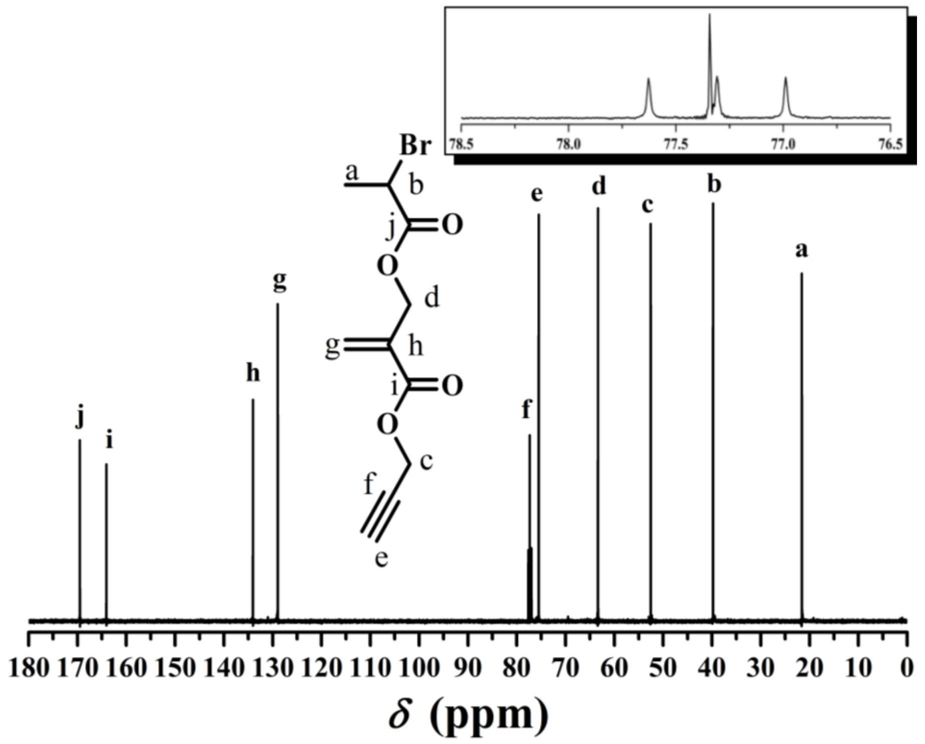

Supplementary Figure 3. ^{13}C NMR spectrum of Br-acrylate-alkyne **3** monomer in $CDCl_3$.

Supplementary Table 1. RAFT Homopolymerization of Br-acrylate-alkyne **3**[a]

Entry	[**3**]:[CDB]:[AIBN]	$M_{n,GPC}$[b] (g/mol)	M_w/M_n[b]	DP[c]	$M_{n,NMR}$[d] (g/mol)
**4a**	60:3:1	3,100	1.28	11	3,200
**4b**	90:3:1	7,900	1.30	21	6,000

[a] Polymerization temperature: 70°C, polymerization time: 20 h, solvent: toluene. [b] Measured by GPC at 35°C in THF. [c] DP represents the degree of polymerization of monomer **3** obtained from ^{1}H NMR, DP = $5S_c/S_{phenyl}$ (S_c and S_{phenyl} are the integration area of 1 proton of alkynyl at 2.62 ppm and 5 protons of phenyl in the range from 7.15 ppm to 7.36 ppm in Figure S1B, respectively). [d] $M_{n,NMR}$ = DP×275+187, 275 and 187 are the molecular weights of monomer **3** and CTA moiety, respectively.

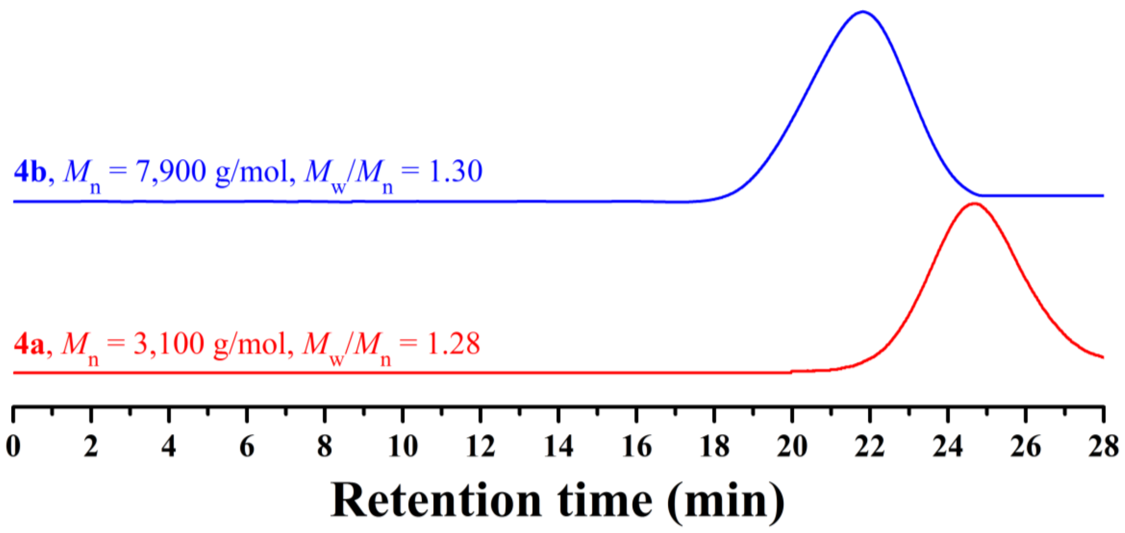

Supplementary Figure 4. GPC traces of PBAA **4** macro-agents in THF.

Supplementary Table 2. CuAAC Reaction between PEO-N_3 and PBAA [a]

Time (h)	S_{PS} [b]	$S_{PEO\text{-}N_3}$ [c]	Reacted PEO-N_3 [d]	D_{graft} [e]
0	1.00	1.13		
1	1.00	0.98	13%	52%
2	1.00	0.91	20%	80%
3	1.00	0.87	23%	92%

[a] PEO-N_3: M_n = 775 g/mol; PBAA **4b**: $M_{n,GPC}$ = 7,900 g/mol, M_w/M_n = 1.30; feeding ratio: [-N_3]:[-C≡CH] = 4:1, [ethyl acrylate]:[-Br] = 150:1, [-C≡CH] = [-Br]; reaction temperature: 60°C. [b] GPC peak area of PS internal standard (M_n = 3,790 g/mol). [c] GPC peak area of unreacted PEO-N_3. [d] The percentage of reacted PEO-N_3 = $1-[(S_{PEO\text{-}N3,t}/S_{PS,t})/(S_{PEO\text{-}N3,0}/S_{PS,0})]$. [e] D_{graft} represents the grafting density of PEO side chain = $\{1-[(S_{PEO\text{-}N3,t}/S_{PS,t})/(S_{PEO\text{-}N3,0}/S_{PS,0})]\}\times([-N_3]:[-C\equiv CH])$.

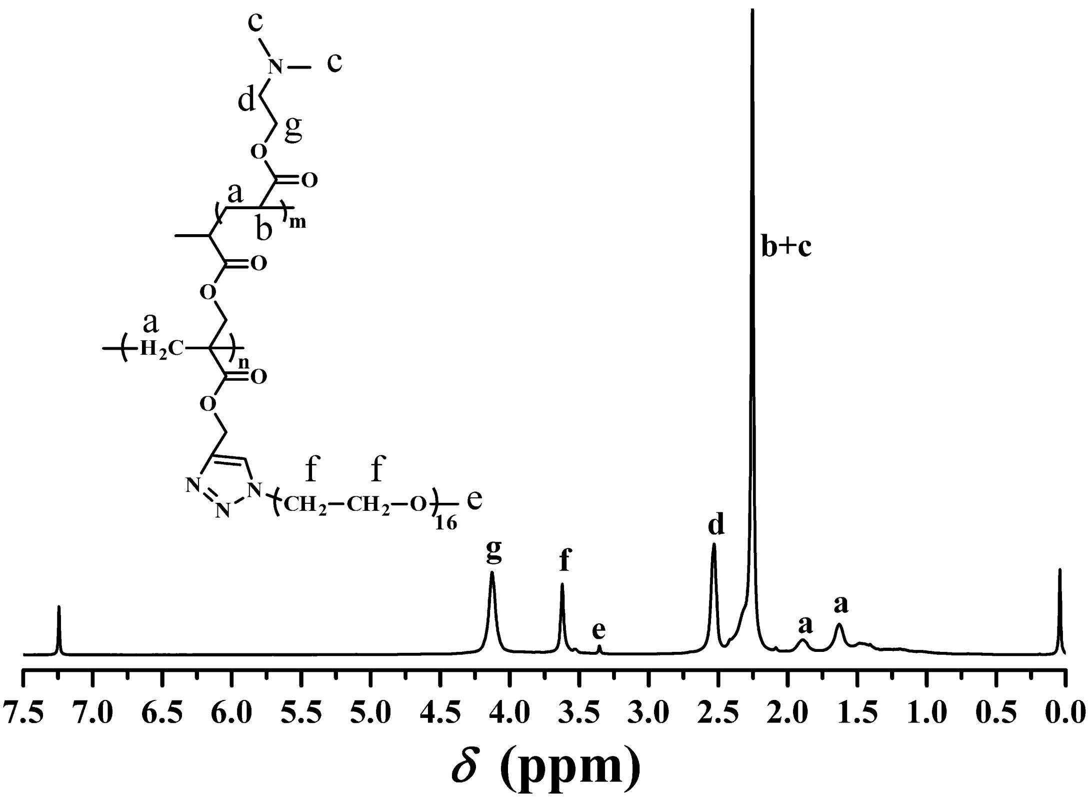

Supplementary Figure 5. ^{1}H NMR spectrum of PA-*g*-PDMAEA/PEO **6** asymmetric molecular double-brush in $CDCl_3$.

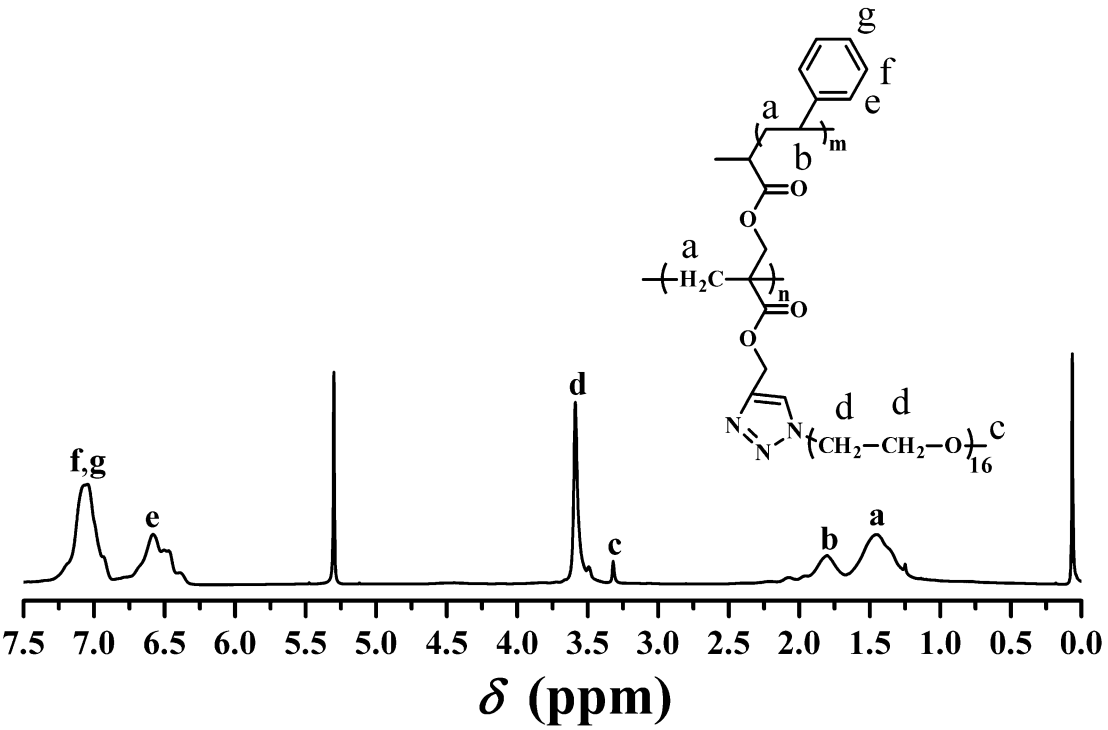

Supplementary Figure 6. ^{1}H NMR spectrum of PA-*g*-PS/PEO **7** asymmetric molecular double-brush in CD_2Cl_2.

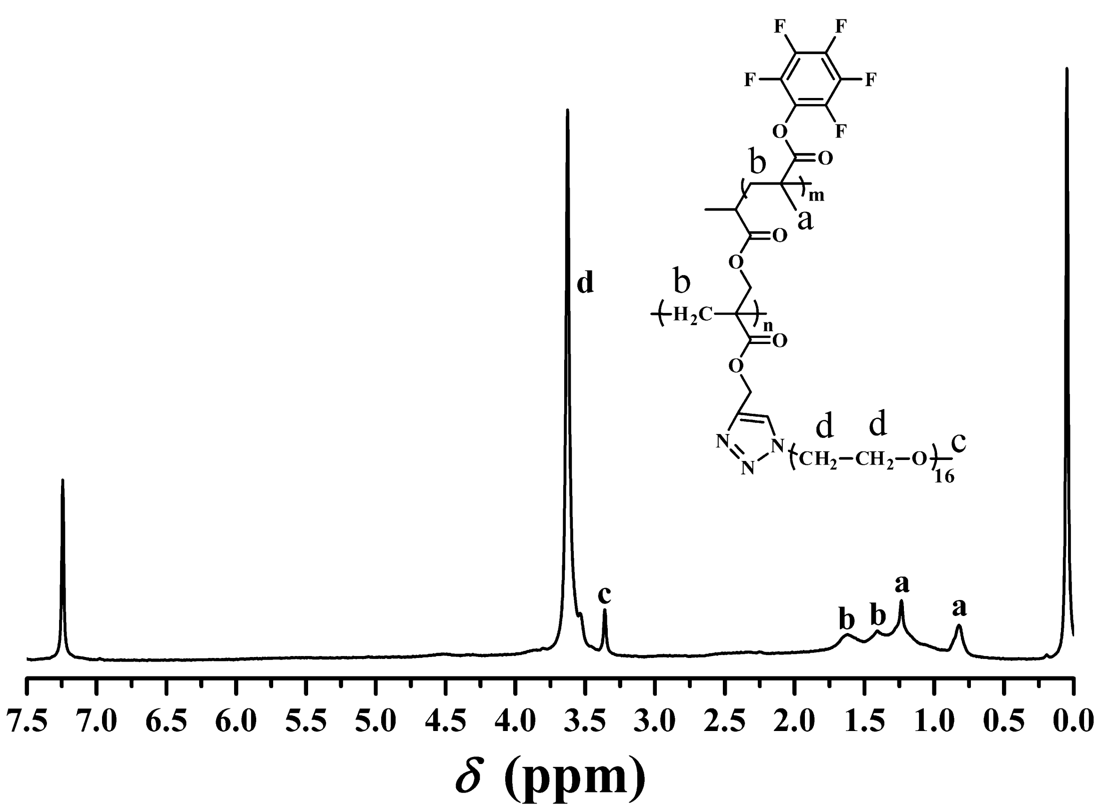

Supplementary Figure 7. ^{1}H NMR spectrum of PA-*g*-PPFMA/PEO **8** asymmetric molecular double-brush in $CDCl_3$.

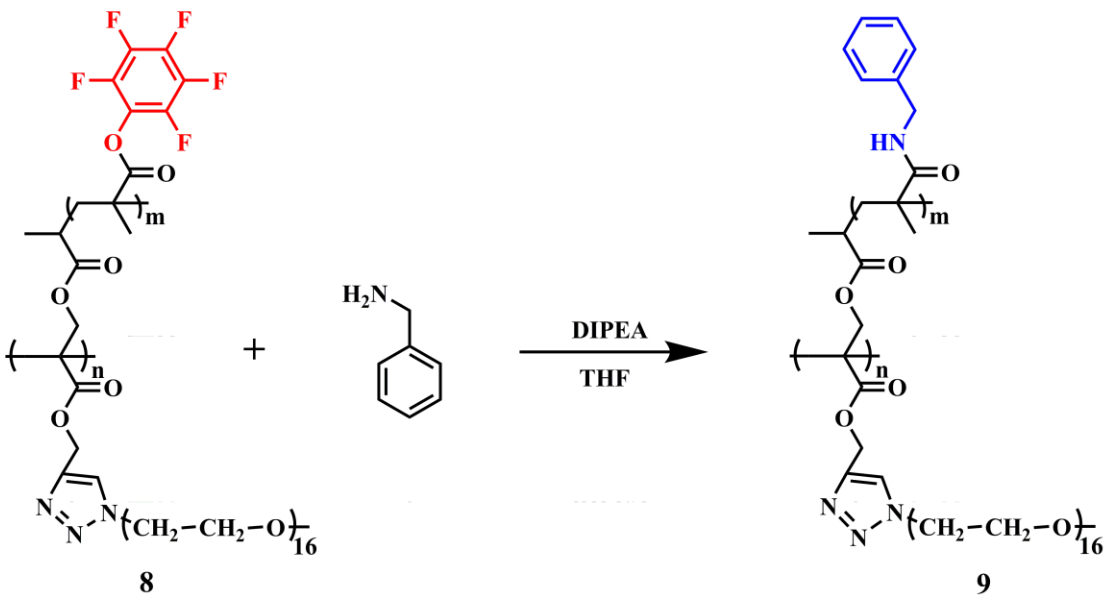

Supplementary Figure 8. Synthesis of PA-*g*-PBMAm/PEO **9** asymmetric molecular double-brush.

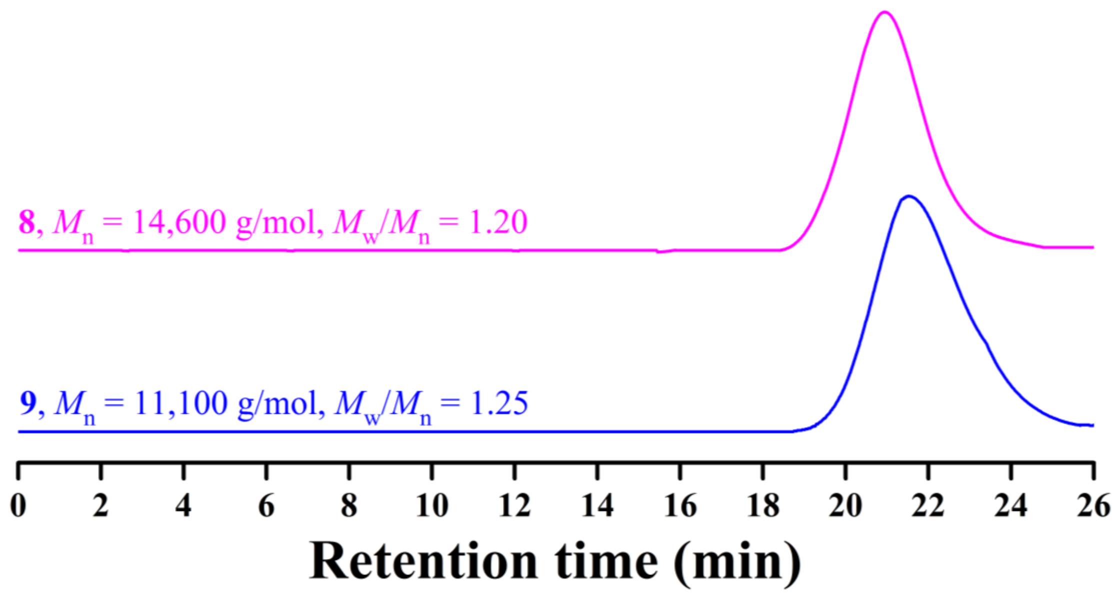

Supplementary Figure 9. GPC traces of PA-*g*-PPFMA/PEO **8** and PA-*g*-PBMAm/PEO **9** asymmetric molecular double-brushes in THF.

Supplementary References

1. Moad, G. *et al*. Living free radical polymerization with reversible addition-fragmentation chain transfer (the life of RAFT). *Polym. Int.* **49,** 993-1001 (2000).
2. Gao, H. & Matyjaszewski, K. Synthesis of molecular brushes by “grafting onto” method: combination of ATRP and click reactions. *J. Am. Chem. Soc.* **129,** 6633-6639 (2007).
3. Eberhardt, M., Mruk, R., Zentel, R. & Theato, P. Synthesis of pentafluorophenyl(meth)acrylate polymers: new precursor polymers for the synthesis of multifunctional materials. *Eur. Polym. J.* **41,** 1569-1575 (2005).
4. Perrier, S., Takolpuckdee, P. & Mars, C. A. Reversible addition-fragmentation chain transfer polymerization: end group modification for functionalized polymers and chain transfer agent recovery. *Macromolecules* **38,** 2033-2036 (2005).
